# Investigating the Structural and Functional Changes in the Optic Nerve in Patients with Early Glaucoma Using the Optical Coherence Tomography (OCT) and RETeval System

**DOI:** 10.3390/s23094504

**Published:** 2023-05-05

**Authors:** Marsida Bekollari, Maria Dettoraki, Valentina Stavrou, Aikaterini Skouroliakou, Panagiotis Liaparinos

**Affiliations:** 1 Department of Biomedical Engineering, University of West Attica, Ag. Spyridonos, 12243 Athens, Greece; mbekolari@uniwa.gr (M.B.);; 2 Department of Ophthalmology, “Elpis” General Hospital, 11522 Athens, Greece

**Keywords:** electroretinography, glaucoma, ophthalmology

## Abstract

The present manuscript introduces an investigation of the structural and functional changes in the optic nerve in patients undergoing glaucoma treatment by comparing optical coherence tomography (OCT) measurements and RETeval system parameters. For such a purpose, 140 eyes were examined at the Ophthalmology Clinic of the “Elpis” General Hospital of Athens between October 2022 and April 2023. A total of 59 out of 140 eyes were from patients with early glaucoma under treatment (case group), 63 were healthy eyes (control group) and 18 were excluded. The experimental measurements were statistically analyzed using the SPSS software package. The main outcomes are summarized below: (i) there was no statistical difference between the right and left eye for both groups, (ii) statistical differences were found between age interval subgroups (30–54 and 55–80 years old) for the control group, mainly for the time response part of the RETeval parameters. Such difference was not indicated by the OCT system, and (iii) a statistical difference occurred between the control and case group for both OCT (through the retinal nerve fiber layer–RNFL thickness) and the RETeval parameters (through the photopic negative response–PhNR). RNFL was found to be correlated to b-wave (ms) and W-ratio parameters. In conclusion, the PhNR obtained by the RETeval system could be a valuable supplementary tool for the objective examination of patients with early glaucoma.

## 1. Introduction

The human eye is a delicate sensory organ, directly exposed to the environment and responsible for various regulations of human functional activities, from seeing things to keeping balance. For this reason, the pathology of the eye (e.g., diabetic retinopathy, cataract, eye cancer, optic nerve or retinal damage, corneal disorders, etc.) can be a significant factor for the degradation of a human’s quality of life [1], including physical, emotional, and social well-being. In particular, more than 1 billion people have vision impairment or blindness from potentially preventable or correctable causes [2]. Nowadays, it is believed that there is a strong connection between well-being visual function and quality of life, constantly increasing and extending the clinical ophthalmology research by taking into account patient measurement outcome assessments [3]. One of the most important eye diseases is glaucoma, which is considered a worldwide leading cause of visual damage and blindness [4,5].

Eye disorder and vision problems due to glaucoma are currently irreversible and leave patients increasingly impaired in daily activities, leading to additional health consequences and dependence on others [6]. Since glaucoma may become a chronic neuropathy in which the optic nerve is damaged, leading to irreversible structural and functional changes in the optic nerve head [7], special treatments and lifelong medications are required [8]. More specifically, gradual loss and degeneration of retinal ganglion cells (RGCs) have been observed, which cause changes in the optic nerve and reduction in RNFL thickness [9]. Although the pathogenesis of glaucoma is not fully understood [10], the major risk factor that conducts glaucoma is considered to be the elevated intraocular pressure (IOP) [5,11]. Consequently, the individuals with IOP greater than 21 mmHg, especially if they have other risk factors, must be carefully managed with the appropriate treatment, usually with ocular hypotensive drops [5,12]. The aim of glaucoma treatment is to lower the IOP in an effort to protect the optic nerve and halt the progression of glaucoma, resulting in the maintenance of visual function. Medications available for reducing the IOP in patients with glaucoma include topical β-adrenergic antagonists, carbonic anhydrase inhibitors, cholinergics, α-adrenergic agonists, prostaglandins, prostamides, etc. [13]. 

Glaucoma generally has a slow and asymptomatic progression until the advanced stages. In the event that glaucoma disease is possible, it is essential to detect early functional and structural changes in the eye in order to maintain vision as long as possible [14]. To achieve this aim, multiple tests can be carried out for the detection of structural changes, including slit-lamp examination, fundus photography, scanning laser ophthalmoscopy and OCT [12,15]. In recent years, electroretinography (ERG) has been considered an important functional and objective diagnostic tool to monitor RGC activity [16,17,18,19,20]. In particular, there is a specific extended protocol that describes an extension to the ERG Standard, namely, the PhNR of the light-adapted (LA) flash ERG that provides information about the function of retinal ganglion cells and their axons [18,19]. It has been shown that patients with glaucoma with visual field defects demonstrate pathologic PhNR values [21,22]. However, conventional ERG testing requires laboratory conditions and a patient’s cooperation, which makes the ERG recording challenging. The RETeval ERG system (LKC Technologies Inc.) is a new portable device, which is easy-to-use and non-invasive, and requires a minimal amount of patient cooperation. Until now, the number of studies on the RETeval ERG parameters for glaucoma is limited [23,24]. To the best of our knowledge, the investigation of the correlation between the RETeval system’s parameters and the OCT thickness parameter, and the demonstration of their diagnostic role in Caucasian patients with early glaucoma, has not been reported.

The main objective of the present work is to: (a) study the structural and functional changes that have occurred in patients with early glaucoma under treatment by using OCT and the RETeval ERG system; (b) compare possible correlations with control subjects; (c) and compare the numeral outcomes of electrophysiology with RNFL thickness, the traditionally used diagnostic parameter in glaucoma. Analytical information regarding the geometry of our experimental workflow is provided in the following section. The significance of our work is to show the impact of electroretinography in monitoring subjects with early glaucoma on future prospects, including examining in a clinical routine, especially with the portable device, which is fast, non-invasive, and patient-friendly.

## 2. Materials and Methods

### 2.1. Spectral-Domain OCT Measurements

**Figure 1:**
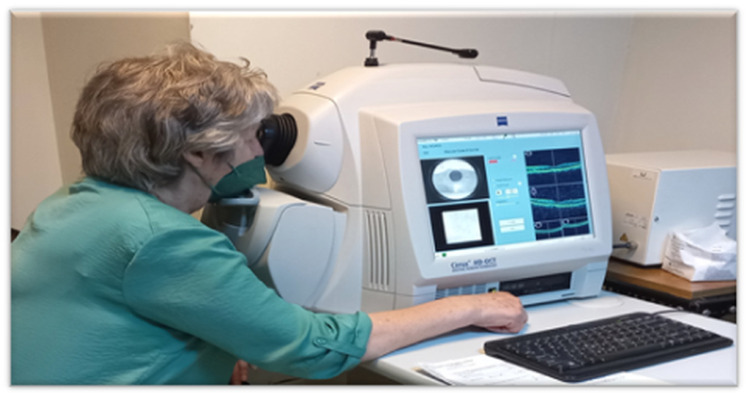
The spectral-domain OCT used in the present study (Cirrus HD-OCT 4000).

The OCT examination was performed using the Cirrus HD-OCT 4000 (Carl Zeiss Meditec Inc., Dublin, CA, USA), as shown in Figure 1. 

All OCT images were obtained by one trained operator (V.S.). Images with signal strength <6, media opacities, and eye movements were excluded from the study. The Optic Disc Cube 200 × 200 protocol was used for all participants. The circumpapillary RNFL thickness data on a 3.46 mm-diameter circle centered on the optic disc center were exported by the software and analyzed. The mean circumpapillary RNFL thickness represents the mean thickness of a 360 degree area around the optic nerve head. 

### 2.2. The ERG Protocol Specifications 

**Figure 2:**
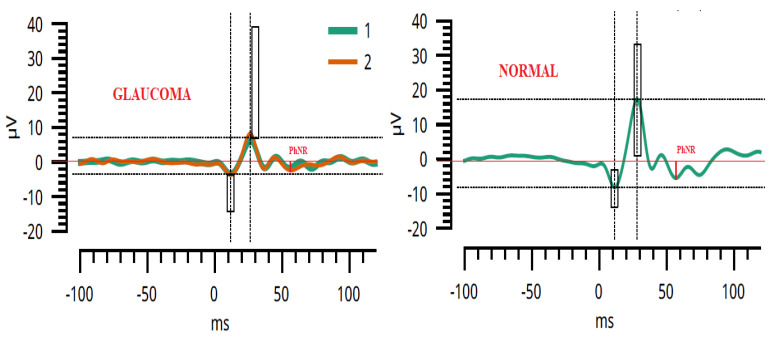
This illustrates the measurement of the PhNR amplitude either from the baseline to the PhNR trough (BT) or from the b-wave peak to the PhNR trough (PT). The image on the left side shows the PhNR signal, indicating glaucoma pathology, while the image on the right side indicates normal functionality. The green line shows the first measurement and the red line represents the second measurement made on the same eye, in order to verify the repeatability of the system.

The PhNR is a slow negative-going wave after the b-wave in response to a brief flash. A reduced PhNR is correlated with retina disorders, including glaucoma or other forms of optic neuropathy [15,18,19,21,25,26]. PhNR measurements can be derived in several different ways, either as the negative trough following the b-wave or at a fixed time point. In the latter case, it is obtained either as a raw amplitude or as a ratio relative to other components of the ERG response [15,18,22,25,27,28]. A PhNR comparison between glaucoma and normal behavior is illustrated in Figure 2. According to the International Society for Clinical Electrophysiology of Vision (ISCEV), the chromatic characteristics of the PhNR protocol include red LED flashes (630–660 nm) on blue LED (450–485 nm) backgrounds. The dynamic range of the red stimulus response generally ranges from 0.01 to 2.0 phot cd s m^−2^ and the background luminance is around 10 phot cd s m^−2^. The recommended range of frequency bandwidth is 0.3–300 Hz [18].

### 2.3. The Portable ERG Device—RETeval

Despite the importance of the PhNR test, the major disadvantage of electrophysiology is its implementation in the daily clinical settings and the huge variability of measurements [27,29,30,31]. A recent development of a handled, portable, and nonmydriatic full-field ERG system has the ability to vanquish some of the limitations considering the variability and difficulty of the implantation. The portable ERG device named RETeval is commercially available from LKC Technologies Inc. and it can operate the ISCEV standard full-field ERG protocols [23,27,32,33,34]. RETeval uses a self-adhering skin sensor strip electrode which simplifies the PhNR recordings in the clinical environment [27,33,35]. The measurements can be performed without mydriasis [33,36]. The device delivers a stimulus with constant retinal illuminance (photopic Td-s) by adjusting the luminance (photopic cd-s/m^2^) to compensate for changes in the pupillary area (mm^2^) [36,37]. The RETeval system and the sensor strip’s electrode were used following the instructions provided by the manufacturer. The protocol PhNR 3.4 Hz Td Long consists of 200 flashes and each set lasts about 60 s. The protocol specifications are briefly summarized in Table 1. More details are analytically provided in Section 2.2. 

### 2.4. Patients, Examination Procedure, and Evaluation

**Figure 3:**
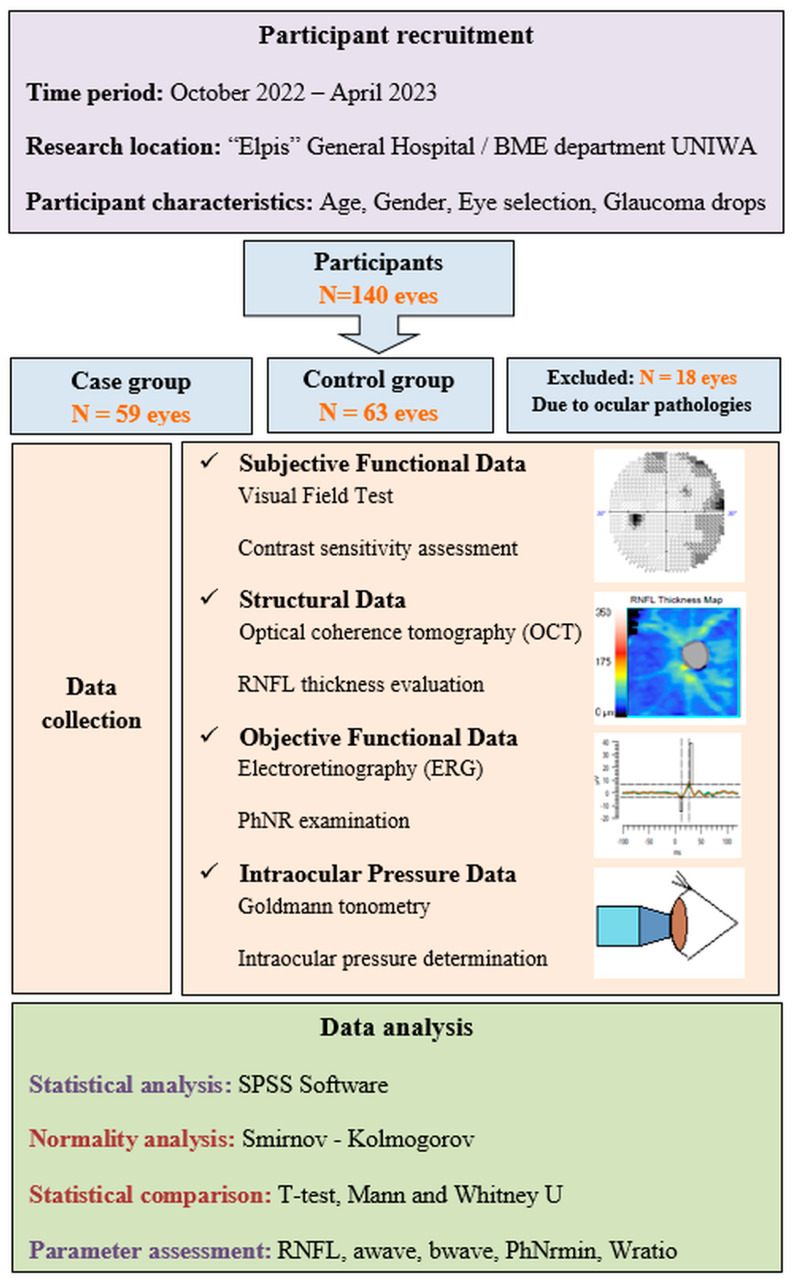
The patient recruitment process, the exclusion criteria, and the workflow of our study.

The patient recruitment process, the exclusion criteria, and the workflow of our study are presented in Figure 3. 

Our study was carried out at the Ophthalmology Clinic of «Elpis» General Hospital in collaboration with the department of Biomedical Engineering of the University of West Attica. The ethics committees of both the University and General Hospital have approved all the procedures used (approval number 82608/19 September 2022), which adhered to the tenets of the Declaration of Helsinki. The patients were examined between October 2022 and April 2023. All participants had been informed about the procedures. Their written informed consent was obtained before the experimental examinations. 

**Figure 4:**
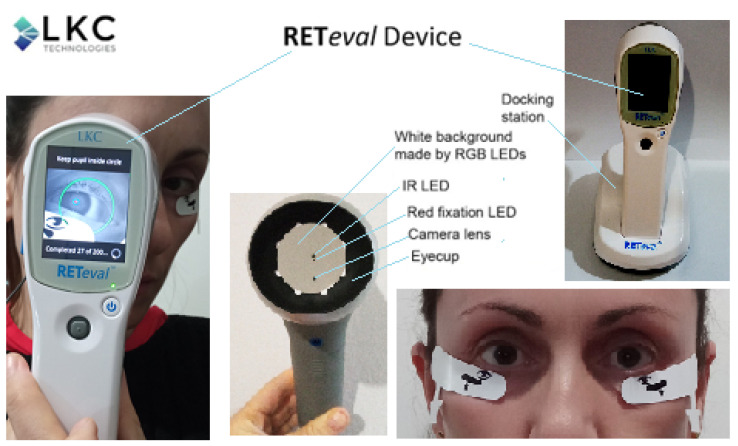
The RETeval recording system used in the present study. The system consists of a hand-held stimulator, a hardware for recording and analysis (7 × 10 × 23 cm, 232 g), a docking station for charging and downloading results to a computer, a soft eye cup, and a disposable skin electrode array (sensor strip) to record the electrical response. The small Ganzfeld dome includes an eyecup, red fixation LED, IR LED, white background, and camera lens. The system also consists of a built-in automated pupillometer. The sensor strip is placed on the orbital rim 2 mm from the margin of the lower eyelid.

Photopic ERG measurements were obtained with the RETeval handheld device (LKC Technologies, Gaithersburg, MD, USA), which uses a series of red flashes of 38 Td-s on a 380-Td blue background. The frequency of the stimuli was 3.4 Hz, and 400 sweeps were averaged for each recording. Light adaption was performed at least 10 min in the clinical testing room prior to the measurement procedure. The RETeval device included a mini Ganzfeld dome with an integrated infrared camera placed at the iris with a 0.08 mm/pixel resolution and 28.3 Hz recording rate, as shown in Figure 4. 

In the center of the dome, a small red fixation spot exists. The pupil size (in mm^2^) is automatically measured in real time during the stimulation. In order to have a constant flash retinal illuminance (Td − s), there was a stimulus luminance adjustment (cd·s/m^2^) based on the relation given below [33]:

Photopic flash retinal illuminance (Td − s) = Photopic flash luminance (cd × s/m^2^) × pupil area (mm^2^) (1)

**Figure 5:**
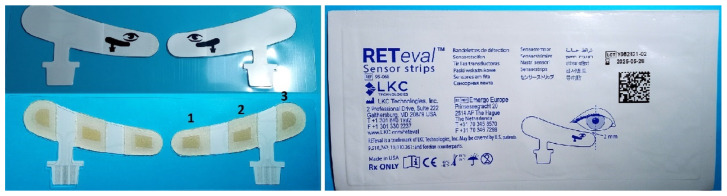
The RETeval sensor strip. The strip contains three electrodes: (1) the active-positive, (2) the reference-negative, and (3) the ground electrode in a single adhesive tape. The image on the right provides the information label of the sensor strips used in the present study.

In order to record the PhNR amplitude, the skin electrode LKC sensor strips, as shown in Figure 5, were placed 2 mm from the margin of the lower eyelid, as illustrated in Figure 4. Before the placement of the electrode, the skin was cleaned with a mildly abrasive gel (NuPrep Skin Prep Gel) to reduce the skin impedance with the sensor strip electrodes. Age-matched reference data were automatically displayed by the RETeval device. The pathological results are colored yellow or red (pathological), while green is used for the normal values.

**Figure 6:**
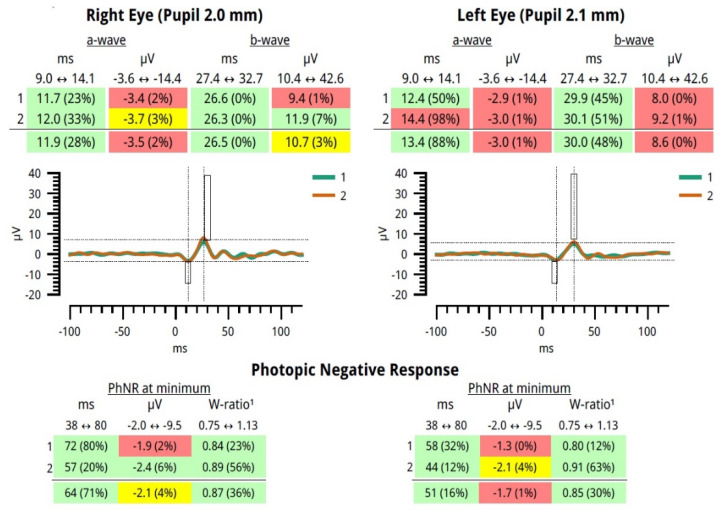
Reported data of our study from the RETeval device using the protocol PhNR 3.4 Hz Td Long.

A specific anonymous experimental measurement example of our data is provided in Figure 6. The pathological numerical values are colored yellow or red, while green corresponds to the normal values.

### 2.5. Parameter Assessment and Statistical Analysis

Ιn the present study, we investigated the following parameters: (i) the a-wave amplitude (μV) and time response (ms), (ii) the b-wave amplitude (μV) and time response (ms), (iii) the minimum (Pmin) PhNR amplitude and implicit time (ms), and (iv) the W-ratio. The reported values were from −100 ms to +120 ms, with the center of the flash at 0 ms. The W-ratio is defined as follows:

W-ratio = (b − pmin)/(b − a)(2)

where a, b and pmin are the voltages relative to baseline, defined as a: a-wave peak, b: b-wave peak, pmin: minimum voltage between 30 ms and 100 ms. The W-ratio is the inverse of PTR (ratio b-wave/PhNR measured from peak-to trough) [5]. Age-matched reference data were automatically displayed by the RETeval device. The output parameters were statistically analyzed and tested using the SPSS software package. All data were checked for normality with the Smirnov–Kolmogorov test. Thereafter, on the data following the normal distribution, statistical comparisons were carried out with a *t*-test, while on the rest of the data, independent samples of Mann–Whitney U tests were performed.

## 3. Results

We enrolled Caucasian patients with early open-angle glaucoma under ocular hypotensive therapy (this is the so-called case group). All participants underwent an ophthalmological examination of both eyes, including slit-lamp biomicroscopy, IOP measured by Goldmann applanation tonometry, visual field (VF) testing, OCT examination, and electroretinography. The VF examination was performed to detect glaucomatous defects with the Zeiss Humphrey Field Analyzer 3 using the Swedish Interactive Threshold Algorithm (SITA) standard test. Glaucomatous VF defects included a nasal step, a generalized depression or hemifield defect, and an inferior or superior paracentral, or Bjerrum‘s scotoma. Participants with a mean deviation (MD) value of ≥−6 dB, which represents early glaucoma, were recruited. All patients had an open-angle glaucoma and were under treatment with topical hypotensive drugs. The control group included healthy individuals with IOP < 21 mmHg, a normal optic nerve head appearance, normal VF test results, a lack of eye drops, or other ocular pathology. In this study, we have included only reliable results, with no artifacts, in the statistical analysis. We examined 140 eyes in total, where 18 eyes were excluded due to the following reasons: high degree of myopia, macular degeneration, low index OCT outcomes. Therefore, we finally examined 59 eyes under treatment (case group) and 63 healthy eyes (control group). In the case group, the patients were treated with topical hypotensive medication, either as a monotherapy or as a combination of drugs, including prostaglandin analogs, alpha-2 agonists, beta-blockers, and carbon anhydrase inhibitors. 

The characteristics of the subjects involved in our study are shown in Table 2. The mean value and the corresponding standard deviation of the following parameters are summarized: (i) age, (ii) RNFL, (iii) a-wave, (iv) b-wave, (v) PhNrmin, (vi) W-ratio, and (vii) IOP. For all subjects under examination, the IOP values were lower than 21 mmHg for both case and control subjects. Here, it is important to mention that all RETeval parameters were examined in both amplitude (μV) and time response (ms) acquisitions, of which characterize the corresponding eye morphological and functional characteristics.

The normality test showed that the parameters found to follow the normal distribution were the RNFL, a-wave, and b-wave for both groups and PhNrmin (μV) for the case group, according to the data provided in Table 3. On the other hand, the rest of the parameters under examination were found not to follow the normal distribution at the 95% confidence level (*p* < 0.05). We performed comparisons on the following evaluating schemes, between (i) the left and right eye for all subjects, and (ii) two different age interval subgroups (30–54 and 55–80) of the control group and (iii) the case and control group.

The comparisons between the left and right eye for all subjects (case and control groups) are provided in Table 4, where no statistically significant differences were found either in the case or the control group. 

**Figure 7:**
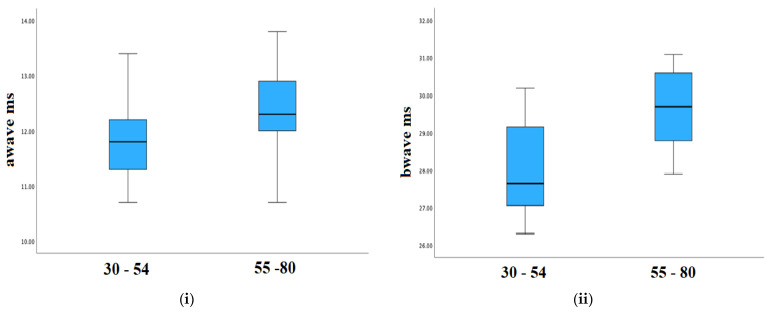
Comparisons of the following parameters: a-wave (ms) (**i**) and b-wave (ms) (**ii**) for the two subgroups with age intervals of 30–54 and 55–80 years old in the control group.

To evaluate whether the measured characteristics depend on age, the control group was divided into two subgroups with age intervals of 30–54 and 55–80 years old. Analytical data of *p*-values are presented in Table 4 for all evaluating parameters. Statistically significant differences were observed between the two subgroups for the a-wave (ms) and the b-wave (ms). In addition, to further examine the difference of the aforementioned parameters, a comprehensive analysis is provided in Figure 7. It was found that the younger subjects appear with lower a-wave (ms) and b-wave (ms) values. More specifically, the following numerical data were obtained for the mean values of (i) a-wave (ms), 11.79 ms for the age interval 30–54 and 12.43 ms for the age interval 55–80; (ii) b-wave (ms), 27.98 ms for the age interval 30–54 and 29.70 ms for the age interval 55–80.

**Figure 8:**
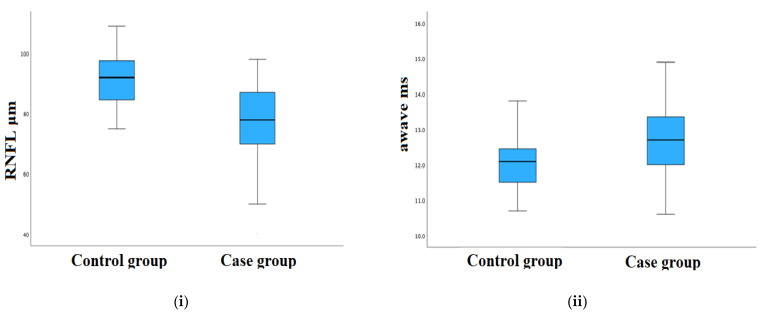

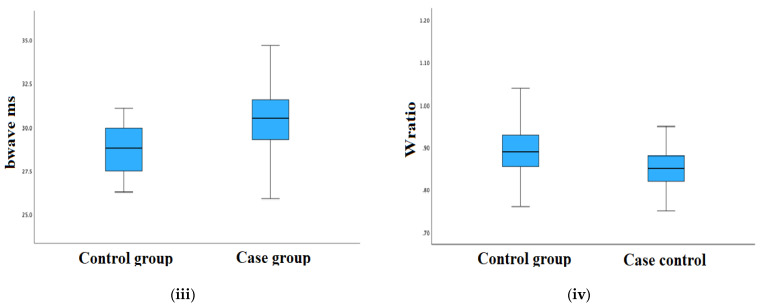
Comparisons of the following parameters: RNFL (μm) (**i**), a-wave (ms) (**ii**), b-wave (ms) (**iii**) and W-ratio (**iv**), between the case and control group for all subjects under investigation.

In order to investigate whether the measured characteristics show discrepancies between the case and control group, analytical statistical comparisons were carried out. The *p*-values of the comparisons are given in Table 4 for all evaluating parameters. We consider it important to provide additional comparisons between the case group (mean age: 67.7 y) and the age subgroup (55–80 years old) of the control group (mean age: 64.4 y); however, no significant differences were observed with respect to the total number of subjects (Table 4), with the exception of a limiting *p*-value of a-wave parameter (0.05). Figure 8 shows an analytical statistical performance of four crucial parameters with the highest statistical difference. In particular, the following numerical data were obtained for the mean value and the corresponding standard deviation of the (i) RFNL (μm), 91.41 ± 8.56 (case group) and 76.63 ± 12.96 (control group); (ii) a-wave (ms), 12.07 ± 0.75 (case group) and 12.76 ± 1.05 (control group); (iii) b-wave (ms), 28.72 ± 1.39 (case group) and 30.53 ± 2.08 (control group); (iv) b-wave (mV), 23.26 ± 6.80 (case group) and 20.77 ± 6.02 (control group); (v) PhNrmin (μs), 58.14 ± 9.69 (case group) and 60.86 ± 9.24 (control group); (vi) PhNrmin (μV), −5.25 ± 1.50 (case group) and −4.37 ± 1.34 (control group); and (vii) W-ratio, 0.897 ± 0.070 (case group) and 0.856 ± 0.055 (control group).

To extend our research to possible correlation of the evaluating parameters, bivariate linear correlation tests with RNFL as the independent parameter were performed. For the case group, RNFL was found to be highly correlated to b-wave (ms) (Pearson corr. = −0.259, sig. = 0.048) and W-ratio (Pearson corr. = −0.373, sig. = 0.004) parameters. For the control group, RNFL was found to be correlated to the W-ratio (Pearson corr. = −0.372, sig. = 0.003) parameter. 

## 4. Discussion

In the current investigation, we assessed the structural changes in the optic nerve in patients with glaucoma by measuring RNFL thickness using the OCT examination, as well as the functional changes in the glaucomatous eyes elicited by the RETeval portable ERG system. Our findings were compared with those of healthy individuals. Moreover, we investigated the relationship of various parameters of the RETeval system with the structural measurements obtained by OCT. The main findings of our study are: (i) no statistical differences occurred in the comparison between the left and right eye for both groups (case and control). This evidence is very important since there is no connection of eye morphological and functional activities with any possible neurotransmission from a specific cerebral hemisphere. In addition, both eyes can be considered for data collection in future glaucoma research, and not only one specific eye, as shown in previous investigations [33]. (ii) Regarding the control group, statistical differences observed in age interval subgroups mainly for the time response parameters were taken by the RETeval system; however, the RNFL parameter obtained from the OCT was not capable of indicating any differences. (iii) The overall comparisons between the case and control group showed differences in both OCT and RETeval parameters. For instance, RNFL was found to be correlated to b-wave (ms) and W-ratio parameters. The aforementioned outcomes may present an important contribution of the RETeval system to glaucoma assessments in combination with OCT measurements.

The diagnostic tests used in our study are objective and noninvasive examinations suitable for the detection of both anatomical and functional changes in the optic nerve of patients with glaucoma. The RETeval system, which became commercially available in 2014, does not require direct corneal or conjunctival contact of the ERG electrode, which makes it technically easier and more comfortable for the patient. Furthermore, it is a portable device and easy-to-use, for which minimal patient cooperation is needed. While the RNFL measurement constitutes a routine examination in patients with glaucoma, the role of PhNR in glaucoma diagnosis and progression is not fully investigated. Recently, several studies have shown that the RhNR seems promising in detecting early glaucoma [22] or in eyes with diagnostic dilemma [8]. In accordance with previous studies, our investigation demonstrated that the PhNRmin amplitude recorded with the RETeval system was significantly reduced in patients with early glaucoma compared to the controls. Moreover, in our study, RNFL thickness was found to be highly correlated to the b-wave and W-ratio parameters in patients with early glaucoma. Until now, no previous study has compared the RETeval system parameters with the OCT thickness parameter in Caucasian patients with early glaucoma and age-matched healthy controls. A previous study, in a Japanese population, observed no significant correlation between any RETeval ERG parameter and OCT thickness either in the healthy control group or in the early glaucoma group [24]. It is widely reported that there are racial differences in optic disc appearance, RNFL thickness and central corneal thickness, which represent factors used for the assessment of glaucomatous damage [38,39]. Therefore, no reliable comparisons can be made between different racial groups regarding the diagnosis and management of glaucoma. Several studies have compared the structural changes observed in glaucoma with the electrophysiology findings recorded with the conventional ERG testing. Cvenkel et al. [8] reported a strong association between the PhNR/b-wave ratio and the peripapillary RNFL thickness in suspected glaucoma eyes. Moreover, the authors demonstrated that in eyes with early glaucoma peripapillary, RNFL thickness was correlated best with the PhNR amplitude and the PERG P50 amplitude. Recently, Awwad et al. [15] found that PhNR amplitudes and PhNR/b-wave ratios were significantly reduced in glaucoma cases compared to the healthy controls, and they showed a significant and progressive decline depending on the severity of glaucoma. Authors also demonstrated that PhNR amplitudes and PhNR/b-wave ratios correlated significantly with standard automated perimetry and OCT parameters in glaucoma. The above studies, however, were conducted in different populations and in different glaucoma severity categories, which may explain the diversity of the results. Interestingly, Machida S et al. [40] observed a linear relationship between PhNR and the RNFL thickness in eyes with open-angle glaucoma, indicating that the inner retinal function declines proportionately with the neural loss in glaucoma.

The small sample size of subjects involved in this research, especially in the control group, may present a limit of the predicted evaluations. Furthermore, the classification of glaucoma in stages (e.g., early/mild, moderate, advanced/severe) could also appear as an additional limitation of the present study. Another factor that could provide important information about the optic disc condition is the inclusion of the fundus examination, which was not taken into consideration. One limitation of our study was the fact that the optic disc appearance was not correlated with the OCT and RETeval findings of glaucomatous and healthy eyes. Furthermore, the examination of the follow-up of the subjects through the investigation of the PhNR ability to monitor the progression over time is still an open issue for research. A future challenge and prospect is the assessment of the duration of the glaucoma treatment as well as the implementation of standard automated perimetry (SAP) data for comparison reasons. All the aforementioned considerations, further evaluating schemes (e.g., the gender influence or the macular ganglion cell–inner plexiform layer thickness), and correlation parameters (e.g., pupil diameter during ERG recordings) may become new fields for future investigations. 

## 5. Conclusions

In this study, we performed a series of experimental measurements in order to indicate the structural and functional changes in the optic nerve in patients with early glaucoma under treatment using the OCT system and electroretinogram taken by the RETeval system. Comparisons were carried out with healthy subjects. Based on the statistical analysis of the experimental data, a summary of the derived findings is given as follows: (a) No differences occurred in the comparison between the left and right eye for both groups (case and control). (b) Statistical differences observed in the age interval subgroups of the control group mainly for the time response parameters were taken by the RETeval system. It is of significance to note that such a difference was not indicated by the OCT system, and (c) the overall comparisons between the case and control group showed differences in both OCT and RETeval parameters, some of which were highly correlated. In conclusion, the PhNR obtained by the RETeval system could be a valuable supplementary tool for the objective examination of patients with early glaucoma.

## Figures and Tables

**Table 1:** The specifications of the PhNR protocol performed with the RETeval device used in the present study.

Description of the Protocol PhNR 3.4 Hz Td Long		
Flash Luminance Energy (Red LED, 621 nm)	Background Luminance (Blue LED, 470 nm)	Flashes
38 Td . s @ 3.4 Hz	380 Td	200

**Table 2:** The characteristics of the subjects involved in this study.

	Mean Value ± Standard Deviation			
Variables	Case Group (*n* = 59)	Control Group (*n* = 63)	Control Group (Age 30–54) (*n* = 36)	Control Group (Age 55–80) (*n* = 27)
Age (y)	67.7 ± 10.3	52.8 ± 12.0	44.1 ± 6.1	64.4 ± 6.8
RNFL (μm)	76.9 ± 12.9	91.4 ± 8.6	91.8 ± 8.9	90.9 ± 8.2
a-wave (μV)	−7.4 ± 2.4	−7.8 ± 2.6	−7.5 ± 2.3	−8.1 ± 3.0
a-wave (ms)	12.8 ± 1.1	12.1 ± 0.8	11.8 ± 0.6	12.4 ± 0.8
b-wave (μV)	20.8 ± 6.0	23.3 ± 6.8	23.2 ± 6.6	23.4 ± 7.2
b-wave (ms)	30.5 ± 2.1	28.7 ± 1.4	28.0 ± 1.3	29.7 ± 1.0
PhNrmin (μV)	−4.4 ± 1.3	−5.2 ± 1.5	−5.1 ± 1.4	−5.4 ± 1.7
PhNrmin (ms)	60.9 ± 9.2	58.1 ± 9.7	56.3 ± 11.6	60.6 ± 5.7
W-ratio	0.86 ± 0.05	0.9 ± 0.1	0.9 ± 0.1	0.9 ± 0.1
IOP (mm Hg)	15.3 ± 2.7	15.6 ± 2.1	15.0 ± 2.1	16.4 ± 1.9

**Table 3:** The normality test of the evaluating parameters of this study.

	Normality Test	
Parameters	Case Group	Control Group
RNFL (μm)	0.200	0.200
a-wave (ms)	0.200	0.200
a-wave (μV)	0.024	0.001
b-wave (ms)	0.200	0.200
b-wave (μV)	0.040	0.019
PhNrmin (ms)	0.005	<0.001
PhNrmin (μV)	0.200	0.014
W-ratio	0.051	0.009

**Table 4:** Table provides *p*-values statistical comparisons between: (i) the left and right eye for all subjects under examination (case and control group), (ii) two different age interval subgroups (30–54 and 55–80) of the control group, (iii) the case and control group for the (a) age group (30–80) and (b) age subgroup (55–80).

	*p*-Values				
	Case Group	Control Group		Case Group/Control	
Parameters	Left and Right Eye	Left and Right Eye	Age 30–54 and 55–80	Age 30–80	Age 55–80
RNFL (μm)	0.497	0.935	0.656	<0.001	<0.001
a-wave (ms)	0.987	0.019	<0.001	<0.001	0.05
a-wave (μV)	1	0.956	0.500	0.601	0.636
b-wave (ms)	0.431	0.816	<0.001	<0.001	0.01
b-wave (μV)	0.481	0.568	0.734	0.037	0.099
PhNrmin (ms)	0.291	0.842	0.067	0.022	0.072
PhNrmin (μV)	0.524	0.536	0.474	0.002	0.009
W-ratio	0.144	0.956	0.775	<0.001	0.007

## Data Availability

Data analysis is contained within the article. Anonymized data presented in this study are available on reasonable request due to the ethical approval statement.
